# Supplementary material for: Cell arrangement impacts metabolic activity and antibiotic tolerance in Pseudomonas aeruginosa biofilms
Source: bioRxiv. 2023 Aug 28:2023.06.20.545666. Originally published 2023 Jun 20. Preprint. [Version 2] doi: 10.1101/2023.06.20.545666 (PMC10462148; doi:10.1101/2023.06.20.545666)
Supplement: Supplement 1 [file media-1.pdf]

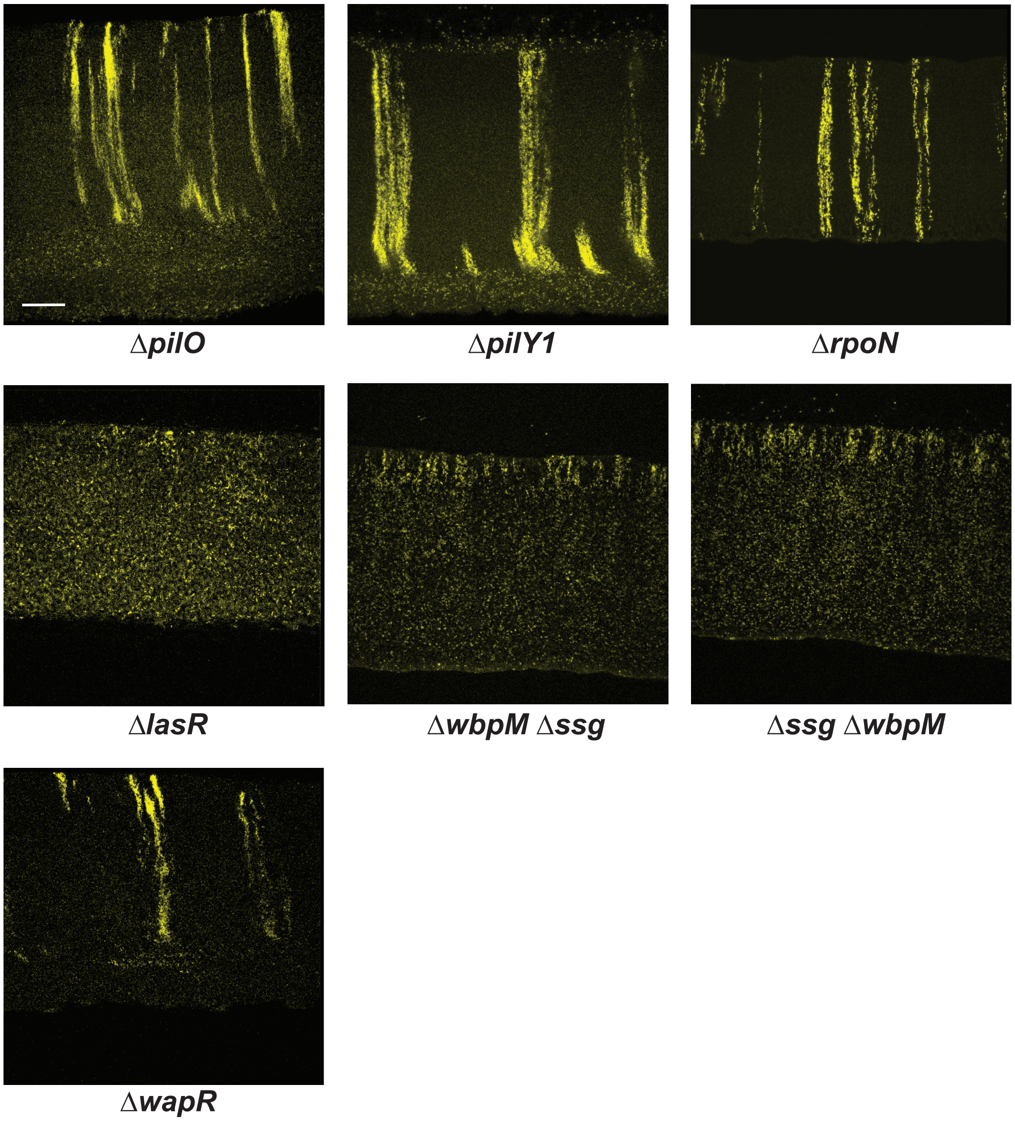

Figure S1.Mutations affecting global regulators, pilus synthesis, and O-antigen synthesis/ attachment alter cell patterning in colony biofilms. Fluorescence micrographs of thin sections from indicated mutant biofilms grown on 1% tryptone and 1% agar for 3 days. The biofilm inocula contained 2.5% of cells that constitutively express mScarlet; 97.5% did not express a fluorophore. Scale bar, 25 μm. Images are representative of at least two independent experiments and mScarlet fluorescence is colored yellow.

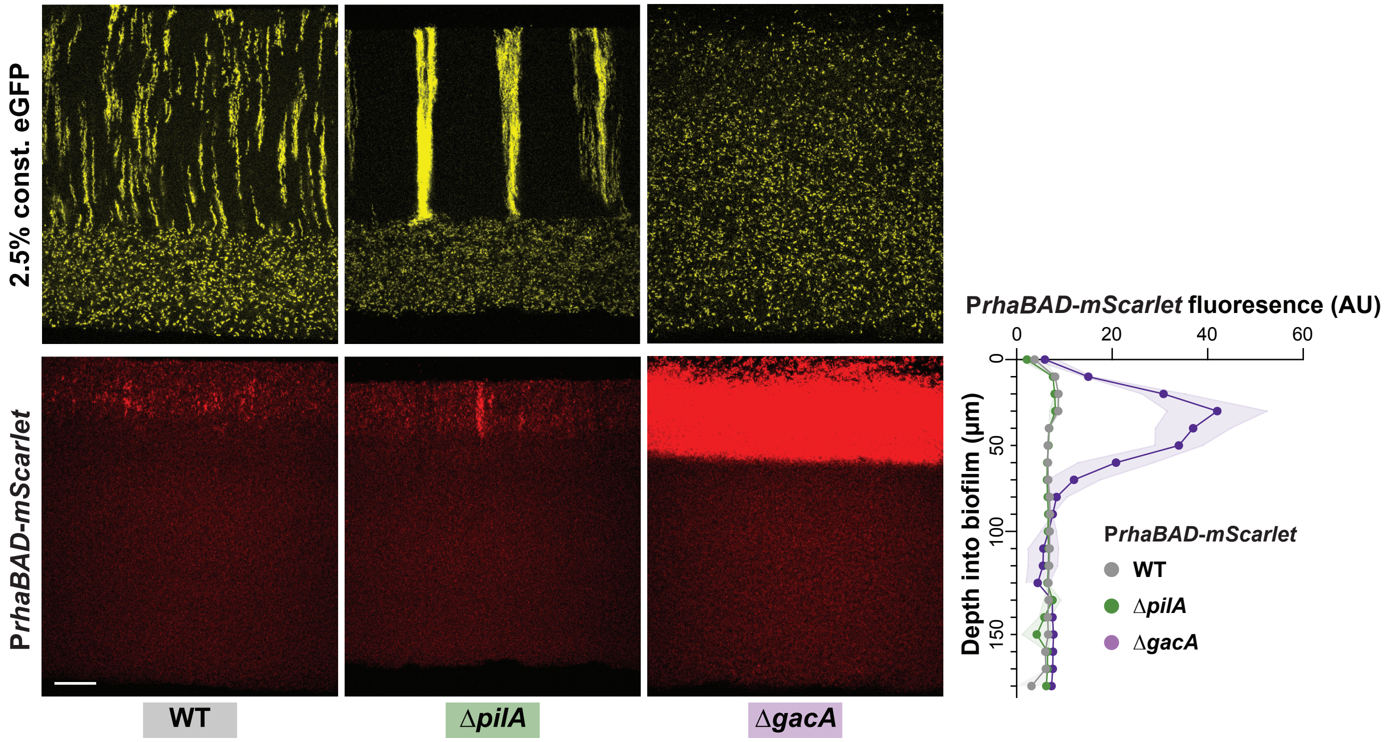

Figure S2. Cellular arrangement affects the uptake and distribution of rhamnose into colony biofilms. Fluorescence micrographs of thin sections from WT and indicated mutant biofilms. Biofilm inocula contained 2.5% cells that constitutively express eGFP and 97.5% RhaSR-*PrhaBAD*-controlled mScarlet-producing strain. Top panels show eGFP fluorescence (colored yellow) and bottom panels show the mScarlet fluorescence for each thin section. Scale bar, 25 μm. Quantification of mScarlet fluorescence is shown on the right.

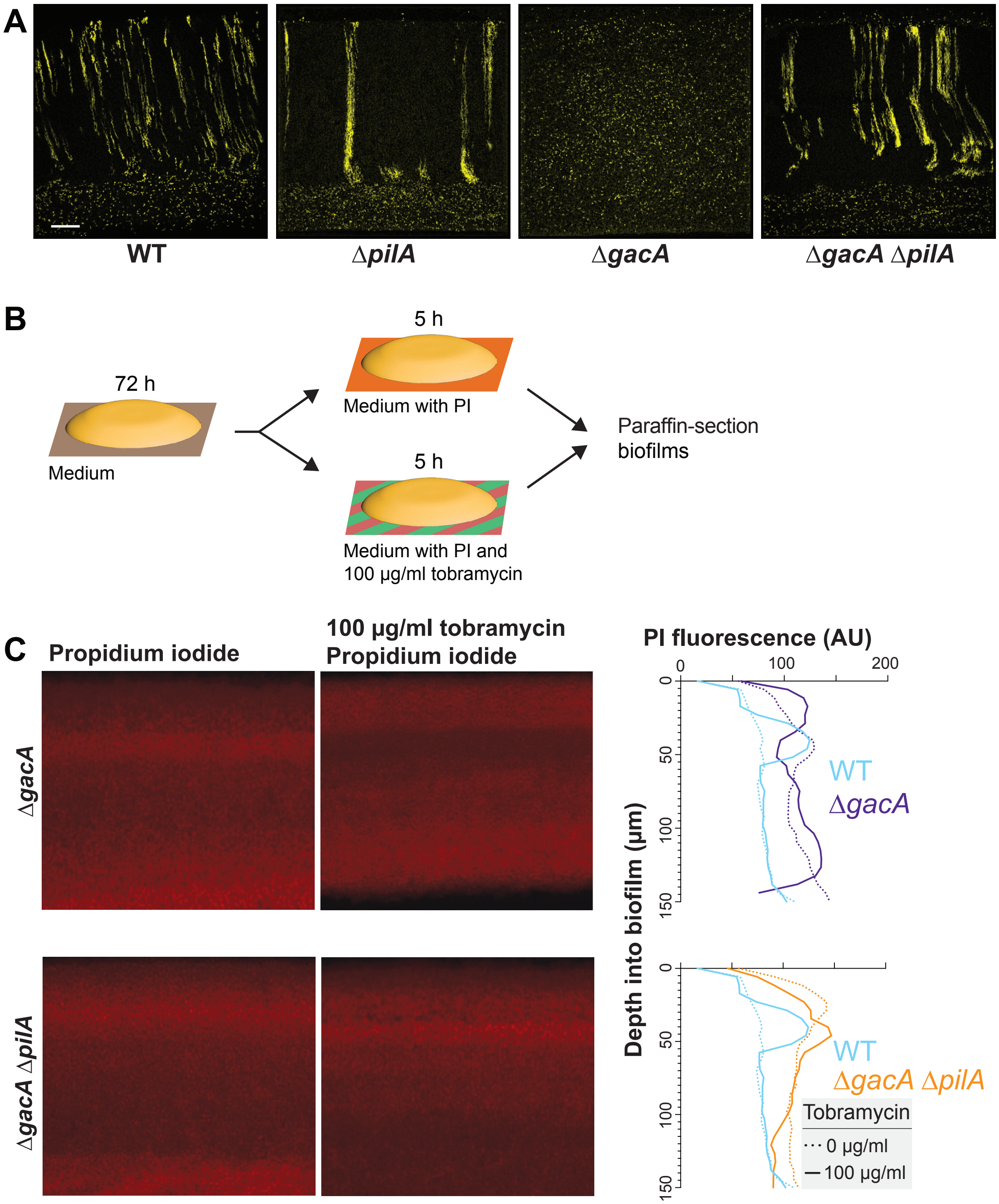

Figure S3. Mutations affecting cellular arrangement alter biofilm antibiotic tolerance profiles. (**A**) Fluorescence micrographs of thin sections from indicated mutant biofilms grown on 1% tryptone and 1% agar for three days. The biofilm inocula contained 2.5% of cells that constitutively express mScarlet (shown as yellow); 97.5% did not express a fluorophore. Scale bar, 25 µm. (**B**) Schematic illustration of the experimental setup for growing *P. aeruginosa* biofilms on agar plates and their subsequent transfer to a medium containing propidium iodide or propidium iodide + tobramycin (**C**) **Left**: Fluorescence micrographs of 3-day-old biofilms exposed to propidium iodide (PI), a DNA stain. **Center**: Fluorescence micrographs of biofilms treated with tobramycin and PI. **Right**: Graphs showing quantification of PI staining. Images shown in this figure are representative of at least two independent experiments.

Table S1. Bacterial strains used in this study.

Number	Strain	Description	Source
Pseudomonas aeruginosa strains			
LD0	UCBPP-PA14 (WT)	Clinical isolate UCBPP-PA14.	[1]
LD4058	PA14 Δ*vfr*	PA14 with *vfr (PA14_08370)* deleted. Made by mating pLD4042 into LD0.	This study
LD732	PA14 Δ*pqsA-C*	PA14 with *pqsA-C (PA14_51430*, *PA14_51420*, and *PA14_51410)* deleted.	[2]
LD1380	PA14 Δ*rhlR*	PA14 with *rhlR (PA14_19120)* deleted. Made by mating pLD1355 into LD0.	[2]
LD4555	PA14 Δ*antABC*	PA14 with *antABC (PA14_32160*, *PA14_32150*, and *PA14_32140) deleted*. Made by mating pLD4537 into LD0.	This study
LD24	PA14 Δ*phz* (also referred to as Δ*phz1/2*)	PA14 with *phz (PA14_09480-PA14_09410* and *PA14_39970-PA14_39880)* operons deleted.	[3]
LD3679	PA14 Δ*gacA*	PA14 with *gacA (PA14_30650)* deleted. Made by mating pLD3079 into LD0.	This study
LD4213	PA14 Δ*lasR*	PA14 with *lasR (PA14_45960)* deleted. Made by mating pLD4228 into LD0.	This study
LD3596	PA14 Δ*aer1*	PA14 with *aer1 (PA14_44300)* deleted. Made by mating pLD3594 into LD0.	This study
LD3604	PA14 Δ*aer2*	PA14 with *aer2 (PA14_02220)* deleted. Made by mating pLD3579 into LD0.	This study
LD3607	PA14 Δ*aer1* Δ*aer2*	PA14 with *aer1* and *aer2 (PA14_44300* and *PA14_02220)* deleted. Made by mating pLD3594 into LD3604.	This study
LD2271	PA14 Δ*nosP*	PA14 with *nosP (PA14_38990 and PA14_38970)* deleted. Made by mating LD2271 into LD0.	This study
LD915	PA14 Δ*anr*	PA14 with *anr* (*PA14_44490)* deleted.	[4]
LD369	PA14 Δ*pilB*	PA14 with *pilB (PA14_58750)* deleted.	[5]
LD1879	PA14 Δ*pilY1*	PA14 with *pilY1 (PA14_60310)* deleted. Made by mating pLD1858 into LD0.	This study

LD3986	PA14 Δ*pilA*	PA14 with *pilA (PA14_58730)* deleted. Made by mating pLD3979 into LD0.	This study
LD4164	PA14 Δ*pilT* Δ*pilU*	PA14 with *pilT pilU (PA14_05180 and PA14_05190)* deleted. Made by mating pLD4137 into LD0.	This study
LD4644	PA14 Δ*ssg*	PA14 with *ssg (PA14_66120)* deleted. Made by mating pLD4630 into LD0.	This study
LD4913	PA14 Δ*wapR*	PA14 with *wapR (PA14_66110)* deleted. Made by mating pLD4832 into LD0.	This study
LD4635	PA14 Δ*wbpM*	PA14 with *wbpM (PA14_23470)* deleted. Made by mating pLD4631 into LD0.	This study
LD3950	PA14 Δ*cheY*	PA14 with *cheY (PA14_45620)* deleted. Made by mating pLD3939 into LD0.	This study
LD371	PA14 Δ*flgK*	PA14 with *flgK (PA14_50360)* deleted.	[5]
LD384	PA14 Δ*pilb* Δ*flgK*	PA14 with *pilb* and *flgK (PA14_58750* and *PA14_5036*0) deleted.	[5]
LD3621	PA14 Δ*motA* Δ*motB*	PA14 with *motA* and *motB* (*PA14_65450* and *PA14_65430)* deleted. Made by mating pLD3629 into LD0.	This study
LD3622	PA14 Δ*motC* Δ*motD*	PA14 with *motC and motD (PA14_45560* and *PA14_45540)* deleted. Made by mating pLD3630 into LD0.	This study
LD1529	PA14 Δ*cupA2*	PA14 with *cupA2 (PA14_37040)* deleted. Made by mating pLD into LD0.	This study
LD1726	PA14 ΔcupA Δ*cupD*	PA14 with *cupD1 (PA14_59710)* deleted. Made by mating pLD into LD0.	This study
LD3070	PA14 Δ*pelA-G*	PA14 with *pelA-G (PA14_24480*, *PA14_24490*, *PA14_24500*, *PA14_24510*, *PA14_24530*, *PA14_24550*, and *PA14_24560)* deleted. Made by mating pLDLD3059 into LD0.	This study
LD3192	PA14 Δ*rpoS*	PA14 with *rpoS (PA14_17480)* deleted. Made by mating pLD3471 into LD0.	[6]
LD3190	PA14 Δ*rpoN*	PA14 with *rpoN (PA14_57940)* deleted. Made by mating pLD3473 into LD0.	[6]
LD3949	PA14 Δ*fliA*	PA14 with *fliA (PA14_45630)* deleted. Made by mating pLD3938 into LD0.	This study

LD3674	PA14 Δ*crc*	PA14 with *crc (PA14_70390)* deleted.	[6]
LD3130	PA14 Δ*ptsP*	PA14 with *ptsP (PA14_04410)* deleted. Made by mating pLD3125 into LD0.	This study
LD3694	PA14 Δ*ptsO*	PA14 with *ptsO* (*PA14_57980)* deleted. Made by mating pLD3635 into LD0.	This study
LD3696	PA14 Δ*ptsO* Δ*ptsP*	PA14 with *ptsO* and *ptsP (PA14_04410* and *PA14_57980)* deleted. Made by mating pLD3635 into LD3130.	This study
LD1888	PA14 Δ*ccoN1* Δ*ccoN2*	PA14 with *ccoN1* and *ccoN2 (PA14_44370* and *PA14_44340)* deleted. Made by mating pLD1610 into LD1784.	[7]
LD1976	PA14 Δ*ccoN1* Δ*ccoN2* Δ*ccoN4*	PA14 with *ccoN1*, *ccoN2*, and *ccoN4 (PA14_443470*, *PA14_44340*, and *PA14_10500)* deleted. Made by mating pLD1264 into LD1888.	[7]
LD1933	PA14 Δ*cco1* Δ*cco2*	PA14 with both *cco* operons *(PA14_44340-PA14_44400*) deleted simultaneously.	[7]
LD3196	PA14 Δ*dipA*	PA14 with *dipA (PA14_66320)* deleted. Made by mating pLD1204 into LD0.	This study
LD4687	PA14 Δ*relA*	PA14 with *relA (PA14_52180)* deleted. Made by mating pLD4642 into LD0.	[8]
LD2177	PA14 Δ*sadC*	PA14 with *sadC (PA14_56280)* deleted. Made by mating pLD2173 into LD0.	[8]
LD2569	PA14 Δ*bifA*	PA14 with *bifA (PA14_56790)* deleted. Made by mating pLD2565 into LD0.	[8]
LD2183	PA14 Δ*roeA*	PA14 with *roeA (PA14_50060)* deleted. Made by mating pLD2179 into LD0.	[8]
LD2227	PA14 Δ*rmcA*	PA14 with *rmcA* (*PA14_07500)* deleted. Made by mating pLD909 into LD0.	[8]
LD2428	PA14 Δ*wspR*	PA14 with *wspR* (*PA14_16500)* deleted. Made by mating pLD4040 into LD0.	[8]
LD3917	PA14 Δ*cyaA*	PA14 with *cyaA (PA14_69610)* deleted. Made by mating pLD3910 into LD0.	This study
LD3920	PA14 Δ*cyaB*	PA14 with *cyaB (PA14_22620)* deleted. Made by mating pLD3911 into LD0.	This study

LD3923	PA14 Δ*cyaA* Δ*cyaB*	PA14 with *cyaA cyaB (PA14_69610* and *PA14_22620)* deleted. Made by mating pLD3910 into LD3920 LD0.	This study
LD3914	PA14 Δ*cpdA*	PA14 with *cpdA (PA14_65690)* deleted. Made by mating pLD3909 into LD0.	This study
LD3625	PA14 Δ*ackA*	PA14 with *ackA (PA14_53470)* deleted. Made by mating pLD3609 into LD0.	This study
LD2729	PA14 Δ*ldhA*	PA14 with *ldhA (PA14_52270)* deleted. Made by mating pLD2728 into LD0.	[9]
LD3646	PA14 Δ*ldhA* Δ*ackA*	PA14 with *ldhA ackA (PA14_53470* and *PA14_52270)* deleted. Made by mating pLD2728 into LD3625.	This study
LD4833	PA14 Δ*pilA* Δ*ssg*	PA14 with *pilA ssg (PA14_58730* and *PA14_66120)* deleted. Made by mating pLD4630 into LD3986.	This study
LD4836	PA14 Δ*pilA* Δ*wbpM*	PA14 with *pilA wbpM (PA14_58730* and *PA14_23470)* deleted. Made by mating pLD4631 into LD3986.	This study
LD4839	PA14 Δ*ssg* Δ*wbpM*	PA14 with *ssg wbpM (PA14_66120* and *PA14_23470)* deleted. Made by mating pLD4631 into LD4644.	This study
LD4835	PA14 Δ*wbpM* Δ*ssg*	PA14 with *wbpM ssg (PA14_23470* and *PA14_66120)* deleted. Made by mating pLD4630 into LD4635.	This study
LD4764	PA14 $P_{PA1/04/03}$-mScarlet	PA14 constitutively expressing mScarlet. Made by mating pLD3433 into LD0.	This study
LD4070	PA14 Δ*vfr* $P_{PA1/04/03}$-mScarlet	PA14 Δ*vfr* constitutively expressing mScarlet. Made by mating pLD3433 into LD4058.	This study
LD4007	PA14 Δ*pqsA-C* $P_{PA1/04/03}$-mScarlet	PA14 Δ*pqsA-C* constitutively expressing mScarlet. Made by mating pLD3433 into LD732.	This study
LD4008	PA14 Δ*rhlR* $P_{PA1/04/03}$-mScarlet	PA14 Δ*rhlR* constitutively expressing mScarlet. Made by mating pLD3433 into LD1380.	This study
LD4765	PA14 Δ*antABC* $P_{PA1/04/03}$-mScarlet	PA14 Δ*antABC* constitutively expressing mScarlet. Made by mating pLD3433 into LD4555.	This study
LD3608	PA14 Δ*phz* (also	PA14Δ*phz* constitutively expressing mScarlet.	This study

	referred to as Δ*phz1/2*) $P_{PA1/04/03}$-mScarlet	Made by mating pLD3433 into LD24.	
LD5074	PA14 Δ*gacA* $P_{PA1/04/03}$-mScarlet	PA14 Δ*gacA* constitutively expressing mScarlet. Made by mating pLD3433 into LD3679.	This study
LD4763	PA14 Δ*lasR* $P_{PA1/04/03}$-mScarlet	PA14 Δ*lasR* constitutively expressing mScarlet. Made by mating pLD3433 into LD4213.	This study
LD5075	PA14 Δ*aer1* $P_{PA1/04/03}$-mScarlet	PA14 Δ*aer1* constitutively expressing mScarlet. Made by mating pLD3433 into LD3596.	This study
LD5076	PA14 Δ*aer2* $P_{PA1/04/03}$-mScarlet	PA14 Δ*aer2* constitutively expressing mScarlet. Made by mating pLD3433 into LD3604.	This study
LD5077	PA14 Δ*aer1* Δ*aer2* $P_{PA1/04/03}$-mScarlet	PA14 Δ*aer1* Δ*aer2* constitutively expressing mScarlet. Made by mating pLD3433 into LD3607.	This study
LD4217	PA14 Δ*nosP* $P_{PA1/04/03}$-mScarlet	PA14 Δ*nosP* constitutively expressing mScarlet. Made by mating pLD3433 into LD.	This study
LD4127	PA14 Δ*anr* $P_{PA1/04/03}$-mScarlet	PA14 Δ*anr* constitutively expressing mScarlet. Made by mating pLD3433 into LD915.	This study
LD5078	PA14 Δ*pilB* $P_{PA1/04/03}$-mScarlet	PA14 Δ*pilB* constitutively expressing mScarlet. Made by mating pLD3433 into LD369.	This study
LD5079	PA14 Δ*pilY1* $P_{PA1/04/03}$-mScarlet	PA14 Δ*pilY1* constitutively expressing mScarlet. Made by mating pLD3433 into LD1879.	This study
LD3987	PA14 Δ*pilA* $P_{PA1/04/03}$-mScarlet	PA14 Δ*pilA* constitutively expressing mScarlet. Made by mating pLD3433 into LD3986.	This study
LD4175	PA14 Δ*pilT* Δ*pilU* $P_{PA1/04/03}$-mScarlet	PA14 Δ*pilT* Δ*pilU* constitutively expressing mScarlet. Made by mating pLD3433 into LD4164.	This study
LD4665	PA14 Δ*ssg* $P_{PA1/04/03}$-mScarlet	PA14 Δ*ssg* constitutively expressing mScarlet. Made by mating pLD3433 into LD4644.	This study
LD4921	PA14 Δ*wapR* $P_{PA1/04/03}$-mScarlet	PA14 Δ*wapR* constitutively expressing mScarlet. Made by mating pLD3433 into LD4913.	This study

LD4666	PA14 Δ*wbpM* $P_{PA1/04/03}$-mScarlet	PA14 Δ*wbpM* constitutively expressing mScarlet. Made by mating pLD3433 into LD4635.	This study
LD3953	PA14 Δ*cheY* $P_{PA1/04/03}$-mScarlet	PA14 Δ*cheY* constitutively expressing mScarlet. Made by mating pLD3433 into LD3950.	This study
LD5080	PA14 Δ*flgK* $P_{PA1/04/03}$-mScarlet	PA14 Δ*flgK* constitutively expressing mScarlet. Made by mating pLD3433 into LD371.	This study
LD5081	PA14 Δ*pilb* Δ*flgK* $P_{PA1/04/03}$-mScarlet	PA14 Δ*pilb* Δ*flgK* constitutively expressing mScarlet. Made by mating pLD3433 into LD384.	This study
LD5082	PA14 Δ*motA* Δ*motB* $P_{PA1/04/03}$-mScarlet	PA14 Δ*motA* Δ*motB* constitutively expressing mScarlet. Made by mating pLD3433 into LD3621.	This study
LD5083	PA14 Δ*motC* Δ*motD* $P_{PA1/04/03}$-mScarlet	PA14 Δ*motC* Δ*motD* constitutively expressing mScarlet. Made by mating pLD3433 into LD3622.	This study
LD5084	PA14 Δ*cupA2* $P_{PA1/04/03}$-mScarlet	PA14 Δ*cupA2* constitutively expressing mScarlet. Made by mating pLD3433 into LD1529.	This study
LD5085	PA14 Δ*cupA* Δ*cupD* $P_{PA1/04/03}$-mScarlet	PA14 Δ*cupA* Δ*cupD* constitutively expressing mScarlet. Made by mating pLD3433 into LD1726.	This study
LD5086	PA14 Δ*pelA-G* $P_{PA1/04/03}$-mScarlet	PA14 Δ*pelA-G* constitutively expressing mScarlet. Made by mating pLD3433 into LD3070.	This study
LD5087	PA14 Δ*rpoS* $P_{PA1/04/03}$-mScarlet	PA14 Δ*rpoS* constitutively expressing mScarlet. Made by mating pLD3433 into LD3192.	This study
LD5088	PA14 Δ*rpoN* $P_{PA1/04/03}$-mScarlet	PA14 Δ*rpoN* constitutively expressing mScarlet. Made by mating pLD3433 into LD3190.	This study
LD3951	PA14 Δ*fliA* $P_{PA1/04/03}$-mScarlet	PA14 Δ*fliA* constitutively expressing mScarlet. Made by mating pLD3433 into LD3949.	This study
LD5089	PA14 Δ*crc* $P_{PA1/04/03}$-mScarlet	PA14 Δ*crc* constitutively expressing mScarlet. Made by mating pLD3433 into LD3674.	This study
LD5090	PA14 Δ*ptsP*	PA14 Δ*ptsP* constitutively expressing mScarlet.	This study

	$P_{PA1/04/03}$-mScarlet	Made by mating pLD3433 into LD3130.	
LD4008	PA14 Δ*ptsO* $P_{PA1/04/03}$-mScarlet	PA14 Δ*ptsO* constitutively expressing mScarlet. Made by mating pLD3433 into LD3694.	This study
LD4009	PA14 Δ*ptsO* Δ*ptsP* $P_{PA1/04/03}$-mScarlet	PA14 Δ*ptsO* Δ*ptsP* constitutively expressing mScarlet. Made by mating pLD3433 into LD3696.	This study
LD2013	PA14 Δ*ccoN1* Δ*ccoN2* $P_{PA1/04/03}$-YFP	PA14 Δ*ccoN1* Δ*ccoN2* constitutively expressing YFP. Made by mating pLD3433 into LD1888.	[7]
LD2136	PA14 Δ*ccoN1* Δ*ccoN2* Δ*ccoN4* $P_{PA1/04/03}$-YFP	PA14 Δ*ccoN1* Δ*ccoN2* Δ*ccoN4* constitutively expressing YFP. Made by mating pLD3433 into LD1976.	[7]
LD2012	PA14 Δ*cco1* Δ*cco2* $P_{PA1/04/03}$-YFP	PA14 Δ*cco1* Δ*cco* constitutively expressing YFP. Made by mating pLD3433 into LD1933.	[7]
LD5091	PA14 Δ*dipA* $P_{PA1/04/03}$-mScarlet	PA14 Δ*dipA* constitutively expressing mScarlet. Made by mating pLD3433 into LD3196.	This study
LD4766	PA14 Δ*relA* $P_{PA1/04/03}$-mScarlet	PA14 Δ*relA* constitutively expressing mScarlet. Made by mating pLD3433 into LD4687.	This study
LD2925	PA14 Δ*sadC* $P_{PA1/04/03}$-YFP	PA14 Δ*sadC* constitutively expressing YFP. Made by mating pLD3433 into LD2177.	This study
LD2773	PA14 Δ*bifA* $P_{PA1/04/03}$-YFP	PA14 Δ*bifA* constitutively expressing YFP. Made by mating pLD3433 into LD2569.	This study
LD2183	PA14 Δ*roeA* $P_{PA1/04/03}$-YFP	PA14 Δ*roeA* constitutively expressing YFP. Made by mating pLD3433 into LD2183.	This study
LD2775	PA14 Δ*rmcA* $P_{PA1/04/03}$-YFP	PA14 Δ*rmcA* constitutively expressing YFP. Made by mating pLD3433 into LD2227.	This study
LD2488	PA14 Δ*wspR* $P_{PA1/04/03}$-YFP	PA14 Δ*wspR* constitutively expressing YFP. Made by mating pLD3433 into LD2428.	This study
LD4069	PA14 Δ*cyaA* Δ*cyaB* $P_{PA1/04/03}$-mScarlet	PA14 Δ*cyaA* Δ*cyaB* constitutively expressing mScarlet. Made by mating pLD3433 into LD3923.	This study
LD4062	PA14 Δ*cpdA* $P_{PA1/04/03}$-mScarlet	PA14 Δ*cpdA* constitutively expressing mScarlet. Made by mating pLD3433 into LD3914.	This study

LD5092	PA14 Δ*ackA* $P_{PA1/04/03}$-mScarlet	PA14 Δ*ackA* constitutively expressing mScarlet. Made by mating pLD3433 into LD3625.	This study
LD5093	PA14 Δ*ldhA* $P_{PA1/04/03}$-mScarlet	PA14 Δ*ldhA* constitutively expressing mScarlet. Made by mating pLD3433 into LD2729.	This study
LD5094	PA14 Δ*pilA* Δ*ssg* $P_{PA1/04/03}$-mScarlet	PA14 Δ*pilA* Δ*ssg* constitutively expressing mScarlet. Made by mating pLD3433 into LD3646.	This study
LD4850	PA14 Δ*pilA* Δ*wbpM* $P_{PA1/04/03}$-mScarlet	PA14 Δ*pilA* Δ*wbpM* constitutively expressing mScarlet. Made by mating pLD3433 into LD4833.	This study
LD4853	PA14 Δ*ldhA* Δ*ackA* $P_{PA1/04/03}$-mScarlet	PA14 Δ*ldhA* Δ*ackA* constitutively expressing mScarlet. Made by mating pLD3433 into LD4836.	This study
LD4852	PA14 Δ*wbpM* Δ*ssg* $P_{PA1/04/03}$-mScarlet	PA14 Δ*wbpM* Δ*ssg* constitutively expressing mScarlet. Made by mating pLD3433 into LD4835.	This study
LD4855	PA14 Δ*ssg* Δ*wbpM* $P_{PA1/04/03}$-mScarlet	PA14 Δ*ssg* Δ*wbpM* constitutively expressing mScarlet. Made by mating pLD3433 into LD4839.	This study
LD3801	WT $P_{PA1/04/03}$-eGFP	PA14 constitutively expressing eGFP. Made by mating pLD3655 into LD0.	This study
LD4292	Δ*pilA* $P_{PA1/04/03}$-eGFP	PA14 constitutively expressing eGFP. Made by mating pLD3655 into LD3986.	This study
LD4842	Δ*wbpM* $P_{PA1/04/03}$-eGFP	PA14 constitutively expressing eGFP. Made by mating pLD3655 into LD4635.	This study
5047	Δ*wbpM* Δ*pilA* $P_{PA1/04/03}$-eGFP	PA14 constitutively expressing eGFP. Made by mating pLD3655 into LD4836.	This study
LD4291	Δ*gacA* $P_{PA1/04/03}$-eGFP	PA14 constitutively expressing eGFP. Made by mating pLD3655 into LD3679.	This study
LD4373	WT attB::rhaSR-PrhaBAD-mScarlet	attB::rhaSR-PrhaBAD-mScarlet. Made by mating pLD4358 into LD0.	This study
LD4374	Δ*gacA* attB::rhaSR-PrhaBAD-mScarlet	attB::rhaSR-PrhaBAD-mScarlet. Made by mating pLD4358 into LD3679.	This study

LD4376	Δ*pilA* attB::rhaSR-PrhaBAD-mScarlet	attB::rhaSR-PrhaBAD-mScarlet. Made by mating pLD4358 into LD3986.	This study
LD4992	Δ*wbpM* attB::rhaSR-PrhaBAD-mScarlet	attB::rhaSR-PrhaBAD-mScarlet. Made by mating pLD4358 into LD4635.	This study
LD5052	Δ*wbpM* Δ*pilA* attB::rhaSR-PrhaBAD-mScarlet	attB::rhaSR-PrhaBAD-mScarlet. Made by mating pLD4358 into LD4836.	This study
E. coli strains			
LD44	UQ950	E. coli DH5α λ(pir) host for cloning; F-Δ(*argF-lac*)169 Φ80 d*lacZ58*(ΔM15) *glnV44*(AS) *rfbD1 gyrA96*(NalR) *recA1 endA1 spoT1 thi-1 hsdR17 deoR* λpir+	D. Lies
LD661	BW29427	Donor strain for conjugation: *thrB1004 pro thi rpsL hsdS lacZ* ΔM15RP4–1360 Δ(*araBAD*)567 Δ*dapA1341*::[*erm pir*(wt)]	W. Metcalf
LD69	β2155	Helper strain. *thrB1004 pro thi strA hsdsS lacZ*ΔM15 (*F' lacZΔM15 lacI^q traΔ36 proA$^+$ proB$^+$*) Δ*dapA::erm* (Erm^r)*pir::*RP4 [*::kan* (Km^r) from SM10]	[10]
LD2901	S17-1	Str^R, Tp^R, F$^-$ RP4-2-Tc::Mu *aphA*::Tn7 *recA* λpir lysogen	R. Simon
Saccharomyces cerevisiae strains			
LD676	InvSc1	*MATα/MATα leu2/leu2 trp1-289/trp1-289 ura3-52/ura3-52 his3-Δ1/his3-Δ1*	Invitrogen

Table S2. Plasmids used in this study.

Plasmid Name	Description	Source
pMQ30	Yeast-based allelic-exchange vector; *sacB$^+$*, CEN/ARSH, URA3$^+$, Gm^R.	[11]
pFLP2	Site-specific excision vector with cI857-controlled FLP recombinase. encoding sequence, sacB$^+$, Amp^R. Used to insert LD2722-based plasmids into P. aeruginosa strains.	[12]

pLD2722	Gm^R, Tet^R flanked by Flp recombinase target (FRT) sites to resolve out resistance cassettes.	[7]
pAKN69-YFP	Gm^R, Cm^R mini-Tn7 $P_{PA1/04/03}$::*yfp*	[13]
pAKN69-mScarlet	Gm^R, Cm^R mini-Tn7 $P_{PA1/04/03}$::*mScarlet*	This study
pLD4042	Δ*vfr (PA14_08370)* PCR fragment introduced into pMQ30 by gap repair cloning in yeast strain InvSc1.	This study
pLD4537	Δ*antABC (PA14_32160*, *PA14_32150*, and *PA14_32140)* PCR fragment introduced into pMQ30 by gap repair cloning in yeast strain InvSc1.	This study
pLD3079	Δ*gacA (PA14_30650)* PCR fragment introduced into pMQ30 by gap repair cloning in yeast strain InvSc1.	This study
pLD4228	Δ*lasR (PA14_45960)* PCR fragment introduced into pMQ30 by gap repair cloning in yeast strain InvSc1.	This study
pLD3594	Δ*aer1 (PA14_44300)* PCR fragment introduced into pMQ30 by gap repair cloning in yeast strain InvSc1.	This study
pLD3579	Δ*aer2 (PA14_02220)* PCR fragment introduced into pMQ30 by gap repair cloning in yeast strain InvSc1.	This study
pLD3594	Δ*aer1* Δ*aer2 (PA14_44300* and *PA14_02220)* PCR fragment introduced into pMQ30 by gap repair cloning in yeast strain InvSc1.	This study
pLD3979	Δ*pilA (PA14_58730)* PCR fragment introduced into pMQ30 by gap repair cloning in yeast strain InvSc1.	This study
pLD4137	Δ*pilT* Δ*pilU (PA14_05180 and PA14_05190)* PCR fragment introduced into pMQ30 by gap repair cloning in yeast strain InvSc1.	This study
pLD4630	Δ*ssg (PA14_66120)* PCR fragment introduced into pMQ30 by gap repair cloning in yeast strain InvSc1.	This study
pLD4832	Δ*wapR (PA14_66110)* PCR fragment introduced into pMQ30 by gap repair cloning in yeast strain InvSc1.	This study
pLD4631	Δ*wbpM (PA14_23470)* PCR fragment introduced into pMQ30 by gap repair cloning in yeast strain InvSc1.	This study
pLD3939	Δ*cheY (PA14_45620)* PCR fragment introduced into pMQ30 by gap repair cloning in yeast strain InvSc1.	This study
pLD3629	Δ*motA* Δ*motB* (*PA14_65450* and *PA14_65430)* PCR fragment introduced into pMQ30 by gap repair cloning in yeast strain	This study

	InvSc1.	
pLD3630	Δ*motC* Δ*motD (PA14_45560* and *PA14_45540)* PCR fragment introduced into pMQ30 by gap repair cloning in yeast strain InvSc1.	This study
pLD3938	Δ*fliA (PA14_45630)* PCR fragment introduced into pMQ30 by gap repair cloning in yeast strain InvSc1.	This study
pLD3635	Δ*ptsP (PA14_04410)* PCR fragment introduced into pMQ30 by gap repair cloning in yeast strain InvSc1.	This study
pLD1204	Δ*dipA (PA14_66320)* PCR fragment introduced into pMQ30 by gap repair cloning in yeast strain InvSc1.	This study
pLD3910	Δ*cyaA PA14_69610* PCR fragment introduced into pMQ30 by gap repair cloning in yeast strain InvSc1.	This study
pLD3909	Δ*cpdA (PA14_65690)* PCR fragment introduced into pMQ30 by gap repair cloning in yeast strain InvSc1.	This study
pLD3609	Δ*ackA (PA14_53470)* PCR fragment introduced into pMQ30 by gap repair cloning in yeast strain InvSc1.	This study
pLD4358	pSEK109-rhaSR-PrhaBAD	This study

Table S3. Primers used in this study.

Primer Number	Sequence
Primers for plasmid pLD4042 (used to make Δ*vfr*)	
3611	cctgcaggtcgactctagagGGGAGAAGATGGACGAACTG
3612	CTACACCGCAAAGAGCACCCTGGTGCATGTGAAAGGAAA
3613	TTTCCTTTCACATGCACCAGGGTGCTCTTTGCGGTGTAG
3614	cagctatgaccatgattacg GATGGCGATTGTGGTTTGTT
Primers for plasmid pLD4537 (used to make Δ*antABC*)	
3962	ggcaaattctgttttatcagaccgcttctgcgttctgatTCAGTCCCTTGATCAGCGC
3963	GTCGAAATGCTCGTGCAGGTACAGCTCCGGTTCGGTGAAC
3964	GTTCACCGAACCGGAGCTGTACCTGCACGAGCATTTCGAC
3965	aattgtgagcggataacaatttcacacaggaaacagctCCCGGCGAATTTCTACTACGA

Primers for plasmid pLD3079 (used to make Δ*gacA*)	
1932	ccaggcaaattctgttttatcagaccgcttctgcgttctgatAGCGTGCGACGTTATGTCT
1933	gaagatgcggtagcgatagACCACTTGCAAGCCTTCG
1934	cgaaggcttgcaagtggtCTATCGCTACCGCATCTTC
1935	ggaattgtgagcggataacaatttcacacaggaaacagctTCACGTTGACGATCAACTCC
Primers for plasmid pLD4228 (used to make Δ*lasR*)	
3813	cctgcaggtcgactctagagACAGGTCCCCGTCATGAAAC
3814	GTTTTCTTGAGCTGGAACGCATGGCCGTTAATTTGGGTCT
3815	AGACCCAAATTAACGGCCATGCGTTCCAGCTCAAGAAAAC
3816	cagctatgaccatgattacgGATCAACATGGTCACCTCCA
Primers for plasmid pLD3594 (used to make Δ*aer1*)	
2958	aggcaaattctgttttatcagaccgcttctgcgttctgatTGGAACCGAAGGACTTCAAC
2942	CTCGTCGGAGGTCTGCTCTTTGAGATTGGTGGTGGTGA
2943	TCACCACCACCAATCTCAAAGAGCAGACCTCCGACGAG
2944	ggaattgtgagcggataacaatttcacacaggaaacagctAGATCCTCTGCGGCGATT
Primers for plasmid pLD3579 (used to make Δ*aer2*)	
2950	aggcaaattctgttttatcagaccgcttctgcgttctgatCACGGTGACGATCACCAGGT
2951	CTCCAGTCGTTCGCCGACAGCGTGGTCCAGCTCGCC
2952	GGCGAGCTGGACCACGCTGTCGGCGAACGACTGGAG
2953	ggaattgtgagcggataacaatttcacacaggaaacagctCTGGCAACTGGTGTTCATGC
Primers for plasmid pLD3979 (used to make Δ*pilA*)	
3583	aggcaaattctgttttatcagaccgcttctgcgttctgatCCTCACCCTCTGAACGAATC
3584	GATTACACCATTCGTGCTCG ATCCAATGCGGTTTCCAGT
3585	ACTGGAAACCGCATTGGAT CGAGCACGAATGGTGTAATC
3586	ggaattgtgagcggataacaatttcacacaggaaacagctACTTCATGGAGCATGCCATC
Primers for plasmid pLD4137 (used to make Δ*pilT* Δ*pilU*)	
3666	aggcaaattctgttttatcagaccgcttctgcgttctgatGAACGCTATGCGGGGTGT
3667	GTCAGGTTCGACAGGTGGTC GGAGAGGTGCAGGTCCGAAG
3668	CTTCGGACCTGCACCTCTCC GACCACCTGTCGAACCTGAC
3669	ggaattgtgagcggataacaatttcacacaggaaacagctGCCTATCCTGTAGGCTGGTG
Primers for plasmid pLD4630 (used to make Δ*ssg*)	

4193	aggcaaattctgttttatcagaccgcttctgcgttctgatAGAAAAACAACCGCGCAAT
4194	GGCTACTTCCGCAAGCACGCCGCTGTCGGTCAGAGTATC
4195	GATACTCTGACCGACAGCGGCGTGCTTGCGGAAGTAGCC
4196	ggaattgtgagcggataacaatttcacacaggaaacagctTACCTGAACCTGACGGGACT
Primers for plasmid pLD4832 (used to make ∆*wapR*)	
4349	aggcaaattctgttttatcagaccgcttctgcgttctgatGGCATGATCGTGGCTTATATC
4350	CAGACGTACCGCAACGTCTCGAACCGGGACAAGGT
4351	ACCTTGTCCCGGTTCGAGACGTTGCGGTACGTCTG
4352	ggaattgtgagcggataacaatttcacacaggaaacagctAAGAATTTCGAGGCGATGG
Primers for plasmid pLD4631 (used to make ∆*wbpM*)	
4201	aggcaaattctgttttatcagaccgcttctgcgttctgatCATGGATGGCATTGATGGTA
4202	GTAGTCGTCCTTCTCCACGGCGAAGGCTAGCCAGAGCGATA
4203	TATCGCTCTGGCTAGCCTTCGCCGTGGAGAAGGACGACTAC
4204	ggaattgtgagcggataacaatttcacacaggaaacagctGGTGATCCGCACCAACTATT
Primers for plasmid pLD3939 (used to make ∆*cheY*)	
3424	aggcaaattctgttttatcagaccgcttctgcgttctgat CCCAGAGACGTGTTACTCGAA
3425	TCAACGGCTATGTGGTCAAAGGACTTGGGCTTCACCAATA
3426	TATTGGTGAAGCCCAAGTCCTCAACGGCTATGTGGTCAAA
3427	ggaattgtgagcggataacaatttcacacaggaaacagctGAGCCGTATACGAGGCATTC
Primers for plasmid pLD3629 (used to make ∆*motA* ∆*motB*)	
2994	aggcaaattctgttttatcagaccgcttctgcgttctgatGCATCGAAAAGATCCAGGAA
2995	ATCCAGCCCTTCGAGGTCGTGCAGAAGAAGCTCAACC
2996	GGTTGAGCTTCTTCTGCACGACCTCGAAGGGCTGGAT
2997	ggaattgtgagcggataacaatttcacacaggaaacagctACCTGCTGCTGGACTTCG
Primers for plasmid pLD3630 (used to make ∆*motC* ∆*motD*)	
3002	aggcaaattctgttttatcagaccgcttctgcgttctgatGTAGGACTGCAAGGCCAGTT
3003	ATCATCCTGGCGTTCGTCGGCACGCAAGCTGCAGATACT
3004	AGTATCTGCAGCTTGCGTGCCGACGAACGCCAGGATGAT
3005	ggaattgtgagcggataacaatttcacacaggaaacagctCCGGTCCTGATGTTCTCCT
Primers for plasmid pLD3938 (used to make ∆*fliA*)	
3484	aggcaaattctgttttatcagaccgcttctgcgttctgatGAACTGTTCGATGCGCTTG

3485	CATCGCCTATCACCTGCTCGAGGAGATCGGCGAGGTACTG
3486	CAGTACCTCGCCGATCTCCTCGAGCAGGTGATAGGCGATG
3487	ggaattgtgagcggataacaatttcacacaggaaacagctCATCGTTCCCGCCAATAC
Primers for plasmid pLD3635 (used to make Δ*ptsP*)	
2475	aggcaaattctgttttatcagaccgcttctgcgttctgatCGGCTCGATCACTTCCTGCA
2476	ctgcgggttgtcgaaggtgagCATGGCTTCCTTGACCCGCTG
2477	cagcgggtcaaggaagccatgCTCACCTTCGACAACCCGCAG
2478	ggaattgtgagcggataacaatttcacacaggaaacagctGATGAACTCCTCGCCGCCC
Primers for plasmid pLD1204 (used to make Δ*dipA*)	
2611	ggaattgtgagcggataacaatttcacacaggaaacagctGCAGCAACTGGTGAAGCGC
2612	GCAGCTCGCTGAGGTTCTcgcGCGCCGGAAGCTCGGA
2613	TCCGAGCTTCCGGCGCgcgAGAACCTCAGCGAGCTGCT
2614	ccaggcaaattctgttttatcagaccgcttctgcgttctgatATCTGCAGCTCGCCCAG
Primers for plasmid pLD3910 (used to make Δ*cyaA*)	
3502	cctgcaggtcgactctagagCAGCTCAATCCCTACCCCTA
3503	TAACGCAGATAGTGCAGCGTGTCGAGATCGAGGCTGAGTG
3504	CACTCAGCCTCGATCTCGACACGCTGCACTATCTGCGTTA
3505	cagctatgaccatgattacgAAGGCAAGGTCTCGATCCTC
Primers for plasmid pLD3909 (used to make Δ*cpdA*)	
3494	cctgcaggtcgactctagagGTCGATGACTTCCAGCGAGT
3495	CGTACTGCTGGTGCAGCTTTTCGAAGTGGACTACGACACC
3496	GGTGTCGTAGTCCACTTCGAAAAGCTGCACCAGCAGTACG
3497	cagctatgaccatgattacg CCTGATCGACAAGGACGAG
Primers for plasmid pLD3609 (used to make Δ*ackA*)	
3022	caggcaaattctgttttatcagaccgcttctgcgttctgatCAATCGGCGAATGAACGCCG
3023	GGATCACCAGTACCCGCGGGTGCAGGGGAAACAGGGAGT
3024	ACTCCCTGTTTCCCCTGCACCCGCGGGTACTGGTGATCC
3025	ggaattgtgagcggataacaatttcacacaggaaacagctCGTGCGCTGAGCAGATCG
Primers for plasmid pLD4358 (used to make pSEK109-rhaSR-PrhaBAD)	
3920	gattcgactgcactagtTTAATCTTTCTGCGAATTGAGATGAC
3921	gattcgactgcctcgagTACGACCAGTCTAAAAAGCGC

1. Rahme LG, Stevens EJ, Wolfort SF, Shao J, Tompkins RG, Ausubel FM. Common virulence factors for bacterial pathogenicity in plants and animals. Science. 1995;268: 1899–1902.

2. Recinos DA, Sekedat MD, Hernandez A, Cohen TS, Sakhtah H, Prince AS, et al. Redundant phenazine operons in Pseudomonas aeruginosa exhibit environment-dependent expression and differential roles in pathogenicity. Proc Natl Acad Sci U S A. 2012;109: 19420–19425.

3. Dietrich LEP, Price-Whelan A, Petersen A, Whiteley M, Newman DK. The phenazine pyocyanin is a terminal signalling factor in the quorum sensing network of Pseudomonas aeruginosa. Mol Microbiol. 2006;61: 1308–1321.

4. Lin Y-C, Sekedat MD, Cornell WC, Silva GM, Okegbe C, Price-Whelan A, et al. Phenazines regulate Nap-dependent denitrification in Pseudomonas aeruginosa biofilms. J Bacteriol. 2018. doi:10.1128/JB.00031-18

5. Hölscher T, Bartels B, Lin Y-C, Gallegos-Monterrosa R, Price-Whelan A, Kolter R, et al. Motility, Chemotaxis and Aerotaxis Contribute to Competitiveness during Bacterial Pellicle Biofilm Development. J Mol Biol. 2015;427: 3695–3708.

6. Evans CR, Smiley MK, Thio SA, Wei M, Price-Whelan A, Min W, et al. Spatial heterogeneity in biofilm metabolism elicited by local control of phenazine methylation. bioRxiv. 2023. doi:10.1101/2023.02.15.528762

7. Jo J, Cortez KL, Cornell WC, Price-Whelan A, Dietrich LE. An orphan cbb3-type cytochrome oxidase subunit supports Pseudomonas aeruginosa biofilm growth and virulence. Elife. 2017;6. doi:10.7554/eLife.30205

8. Okegbe C, Fields BL, Cole SJ, Beierschmitt C, Morgan CJ, Price-Whelan A, et al. Electron-shuttling antibiotics structure bacterial communities by modulating cellular levels of c-di-GMP. Proc Natl Acad Sci U S A. 2017;114: E5236–E5245.

9. Lin Y-C, Cornell WC, Jo J, Price-Whelan A, Dietrich LEP. The Pseudomonas aeruginosa Complement of Lactate Dehydrogenases Enables Use of d- and l-Lactate and Metabolic Cross-Feeding. MBio. 2018;9. doi:10.1128/mBio.00961-18

10. Dehio C, Meyer M. Maintenance of broad-host-range incompatibility group P and group Q plasmids and transposition of Tn5 in Bartonella henselae following conjugal plasmid transfer from Escherichia coli. J Bacteriol. 1997;179: 538–540.

11. Shanks RMQ, Caiazza NC, Hinsa SM, Toutain CM, O'Toole GA. Saccharomyces cerevisiae-based molecular tool kit for manipulation of genes from gram-negative bacteria. Appl Environ Microbiol. 2006;72: 5027–5036.

12. Hoang TT, Karkhoff-Schweizer RR, Kutchma AJ, Schweizer HP. A broad-host-range Flp-FRT recombination system for site-specific excision of chromosomally-located DNA sequences: application for isolation of unmarked Pseudomonas aeruginosa mutants. Gene. 1998;212: 77–86.

13. Lambertsen L, Sternberg C, Molin S. Mini-Tn7 transposons for site-specific tagging of bacteria with fluorescent proteins. Environ Microbiol. 2004;6: 726–732.
